# PCSK-9-inhibitor therapy improves endothelial function in high-risk patients with cardiovascular disease

**DOI:** 10.1007/s00392-024-02556-6

**Published:** 2024-11-20

**Authors:** Dennis Kannenkeril, Agnes Bosch, Julie Kolwelter, Kristina Striepe, Laura Berner, Robert Pietschner, Christian Ott, Mario Schiffer, Stephan Achenbach, Roland E. Schmieder

**Affiliations:** 1 Department of Nephrology and Hypertension, University Hospital Erlangen, Friedrich-Alexander University Erlangen-Nuremberg (FAU), Ulmenweg 18, 91054 Erlangen, Germany; 2 Department of Cardiology, University Hospital Erlangen, Friedrich-Alexander University Erlangen-Nuremberg (FAU), Erlangen, Germany

**Keywords:** Evolocumab, Endothelial function, PCSK-9 inhibitor, Flow-mediated vasodilation, Vasoactive range

## Abstract

**Background:**

Impaired endothelial function predicts cardiovascular events. The aim of this study was to analyze the effect of evolocumab on endothelial function in patients with cardiovascular disease.

**Methods:**

This was a prospective, double-blinded, randomized, controlled, single center study including patients with cardiovascular disease and treated with statins. Patients were consecutively randomized (1:1) to either evolocumab treatment or placebo. All patients underwent examination of endothelial function at baseline, and after 1, 4 and 8 weeks of treatment by a semi-automatic high-resolution ultrasound system (UNEX EF 18G). Parameters of endothelial function were flow-mediated vasodilation (FMD), low flow-mediated vasoconstriction (L-FMC) and vasoactive range (VAR).

**Results:**

Hundred three patients with a mean age of 66.2 ± 7.7 years and a mean LDL-cholesterol of 98 ± 19.1 mg/dl completed the study. The change in VAR from baseline to week 8 was significantly different with evolocumab compared to placebo (*p* = 0.045). Moreover, VAR increased after 8 weeks of treatment with evolocumab compared to baseline (*p* = 0.034). No change has been noticed in FMD and L-FMC after 8 weeks of treatment with evolocumab.

In subgroup analyses, VAR improved in patients with age ≤ 67 years, lower systolic blood pressure (≤ 125 mmHg) and higher baseline LDL-cholesterol (> 95 mg/dl), (*p* = 0.006, *p* = 0.049 and *p* = 0.042, respectively) after 8 weeks of evolocumab treatment. No serious adverse event related to study medication occurred during the study.

**Conclusion:**

Our data indicate that endothelial function improved with evolocumab treatment in high-risk patients on statin therapy with preexisting cardiovascular disease. Our results contribute to the mechanistic explanation why lower incidence of the cardiovascular composite endpoint has been demonstrated in the FOURIER study.

**Graphical abstract:**

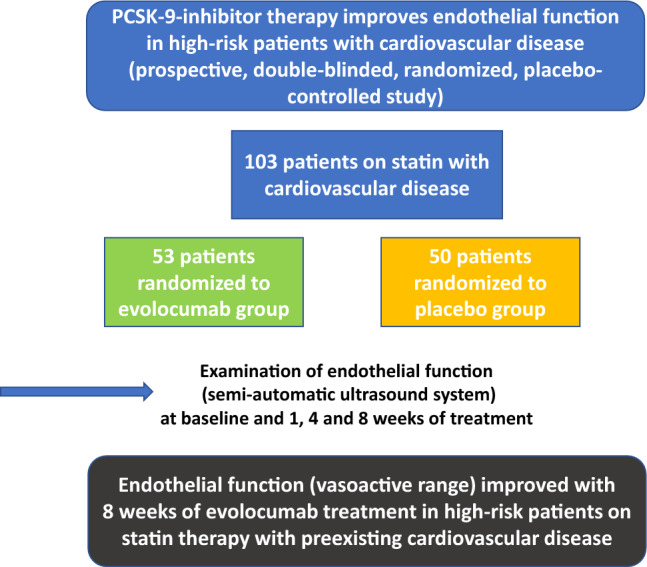

## Introduction

The European Society of Cardiology and the American Heart Association have adapted their guidelines in the management of blood cholesterol based on the motto “the lower the better” [1, 2]. Proprotein convertase subtilisin/kexin type 9 (PCSK-9) inhibition is a new treatment paradigm for hypercholesterolemia and achieves additional LDL reduction by roughly 60% on top of statin therapy [3]. Recently, in a large prospective study (FOURIER) with 27.500 patients at high CV risk evolocumab lowered LDL-C in patients already on optimized lipid lowering therapy including a statin and substantially reduced CV events [3]. However, the mechanisms mediating the improvement of CV outcomes under therapy with evolocumab are unclear at present.

Intact endothelial function is a key component of vascular health and contributes to protective, anti-atherosclerotic effects on the vasculature. At least in part, these protective effects are due to endothelial release of nitric oxide (NO) [4]. Conversely, impaired endothelial function has been linked with increased future CV event rates [5–7] and peripheral endothelial function correlates with coronary artery endothelial function [8]. Endothelial function in humans can be assessed with a well-established and non-invasive method, which has been introduced several years before, by the measurement of flow-mediated vasodilation (FMD), representing vasodilator responsiveness [9]. Recent evidence suggests that concurrent assessment of low flow-mediated vasoconstriction (L-FMC), representing the vasoconstrictor responsiveness, improves characterization of underlying CV and coronary disease compared with each parameter alone [10, 11]. In addition, it has been demonstrated that vasoactive range (VAR), representing total vasomotor responsiveness, has the highest predictive power for CV risk compared to FMD and L-FMC alone [12].

Our aim with this study was to demonstrate that treatment with evolocumab exerts a rapid improvement of endothelial function, even in high risk patients already on optimized lipid lowering therapy including a statin, thereby providing a pathogenic explanation of the effects demonstrated in the FOURIER trial.

## Methods

### Study design

The EVAS trial is a prospective, randomized, double-blind, placebo-controlled, parallel-arm, phase IV clinical trial including patients with CV disease at the Clinical Research Center of the Department of Nephrology and Hypertension, University Hospital Erlangen-Nuremberg, Germany. The study protocol and amendments were submitted and approved at the local ethics committee (application no.: 66_18 Az; University of Erlangen-Nürnberg, Germany) and the study was conducted in accordance with the Declaration of Helsinki and the principles of “good clinical practice” guidelines. The study was registered at www.clinicaltrials.gov (NCT03626831). Patients were simultaneously recruited from investigator’s outpatient clinics, referring physicians, and advertisement in local newspapers. Written informed consent was obtained from each patient before study inclusion. The patients were screened based on the inclusion/exclusion criteria and for feasibility of endothelial function measurement (in particular to ensure that adequate imaging of the brachial artery is possible).

After obtaining anthropometrics, medical history and concomitant medication, physical examination was performed at screening visit. Systolic and diastolic office blood pressure (BP) levels were recorded in a standardized fashion according to guideline recommendations at pre-specified visits of the study [13]. Moreover, blood samples were taken in order to measure glucose, lipid levels, and other biochemical parameters such as renal parameters and liver enzymes.

**Fig. 1:**
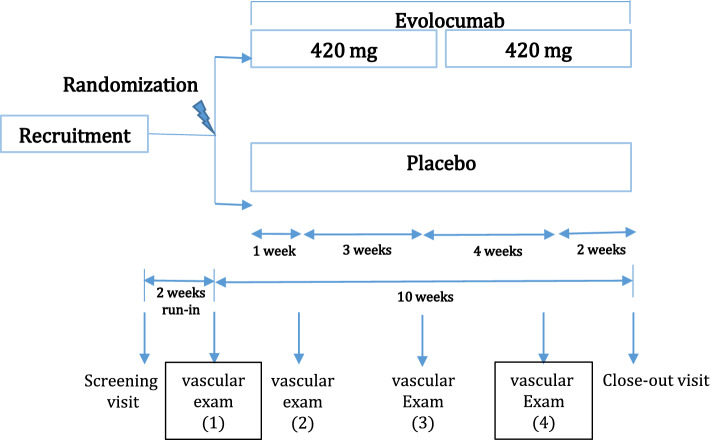
Study design. The EVAS trial is a prospective, randomized, double-blind, placebo-controlled, parallel-arm, phase IV clinical trial. After 2 weeks run-in phase, baseline assessment of endothelial function parameters were performed and immediately thereafter patients were consecutively randomized (1:1) to either SC 420 mg evolocumab treatment or SC placebo. After 4 weeks of randomization, another same dose injection of the study drug (evolocumab/placebo) was administered. After 1, 4 and 8 weeks of treatment, testing of endothelial function were repeated

After 2 weeks run-in phase, baseline assessment of endothelial function parameters were performed and immediately thereafter patients were consecutively randomized (1:1) to either 420 mg subcutaneous (SC) evolocumab treatment or SC placebo (Fig. 1). Four weeks after randomization, a second dose of the study drug (evolocumab/placebo) was administered. After 1, 4 and 8 weeks of treatment, assessment of endothelial function was repeated. Finally, a close out visit (10 weeks after randomization) was performed to gather additional safety information.

The financial supporter AMGEN did not contribute to study conduction, data collection and interpretation of the data.

### Study population

Inclusion criteria (similar to FOURIER study) were male and female patients aged between 40 and 80 years, with clinically evident atherosclerotic CV disease as evidenced by any of the following:

- Diagnosis of coronary artery disease as evidenced by acute coronary syndrome, myocardial infarction, coronary stent implantation, coronary stenosis ≥ 50% by coronary angiography.
- Diagnosis of non-hemorrhagic stroke or transient ischemic attack (TIA).
- Symptomatic peripheral arterial disease, as evidenced by intermittent claudication with ankle-brachial index < 0.85, or peripheral arterial revascularization procedure, or amputation due to atherosclerotic disease, or artery stenosis ≥ 50% by angiography.

Patients had to have a fasting LDL-C level of 70 mg per deciliter (1.8 mmol per liter) or higher or a non-high-density lipoprotein cholesterol (HDL-C) level of 100 mg per deciliter (2.6 mmol per liter) or higher while on optimized regimen of lipid-lowering therapy, which was defined as a high-intensity statin therapy but must have been at least atorvastatin at a dose of 20 mg daily or its equivalent, with or without ezetimibe. Main exclusion criteria were patients with statin intolerance, known homozygous familial hypercholesterolemia, severe renal (eGFR < 30 ml/min/1.73 m^2^) or liver (liver enzymes 3 times above the upper limit of normal) or heart failure (ejection fraction < 30%), atrial fibrillation, known hemorrhagic stroke at any time or patients with myocardial infarction or stroke within previous 4 weeks. Furthermore, recipient of organ transplant, history of malignancy (except non-melanoma skin cancers, cervical in-situ carcinoma, breast ductal carcinoma in situ, or stage 1 prostate carcinoma) within the last 10 years were excluded. In all patients, assessment of endothelial function was performed to check if analysis can be done. Inability to image the brachial artery and to perform endothelial function assessment was another exclusion criteria.

### Assessment of endothelial function parameters

The assessment of endothelial function was performed based on evidence-based recommendations [14]. The protocol of measurement in this study has been described in detail in our former study [15]. Briefly, endothelial function parameters were measured using the semi-automatic UNEX EF device (UNEX EF 18G, UNEX Corp., Nagoya, Japan). This device and it’s method of measurement have been described previously [12, 16]. The device has been found to be reliable across various patient populations and institutions [17]. Patients were informed to fast and abstain from smoking, alcohol, caffeine and antioxidant vitamin supplements on the day of examination to avoid acute influence of these on endothelial function. All measurements were conducted in the morning, with subjects being fasted overnight. During a measurement, the diameter of the brachial artery changes. Three diameters of the brachial artery are obtained: baseline diameter (pre-cuff-inflation diameter), minimum diameter (pre-cuff-deflation diameter) and maximum diameter (post-cuff-deflation diameter). FMD, L-FMC and VAR are calculated from these diameters based on formulas described previously [12].

### Statistics

The primary objective of the study was the change of endothelial function parameters after 8 weeks of treatment with evolocumab compared to placebo treatment. Following new evidence, we have chosen the VAR out of the endothelial function parameters as primary parameter, having highest predictive power for CV risk compared to FMD and L-FMC alone [12]. The secondary objective was the change of endothelial function parameters with respect to baseline. In addition, we performed exploratory analysis of change of endothelial function parameters after 1 week and 8 weeks.

All analyses of this study were performed in patients presenting no major protocol violations. Normal distribution of the parameters was assessed by the Kolmogorov–Smirnov test before further analysis. Continuous variables are presented as mean with SD. Categorical variables are presented as frequency and percentage. The appropriate parametric and non-parametric tests were used to compare the continuous variables. *X*^2^-Test/Fisher’s exact test were used to compare categorical variables. Vascular parameters and patients clinical and laboratory characteristics were analysed by Student’s *t*-test for paired and unpaired (evolocumab vs. placebo) samples, if normally distributed. Non-normally distributed parameters were analysed by Wilcoxon test and Mann–Whitney *U* test, respectively. Correlations were assessed using Spearmann and Pearson correlation coefficient for non-normally and normally distributed variables, respectively. A two-sided *p* value < 0.05 was considered statistically significant. Subgroup analyses were performed only within the evolocumab group based on prespecified subgroups created according to the median of age, baseline LDL-C level, baseline systolic office BP, achieved LDL-C level and HbA1c. Statistical analyses were performed using IBM SPSS Statistics for Windows, Version 28.0.0.0 (IBM Corp., Armonk, NY, USA).

## Results

### Clinical characteristics of the study population

**Fig. 2:**
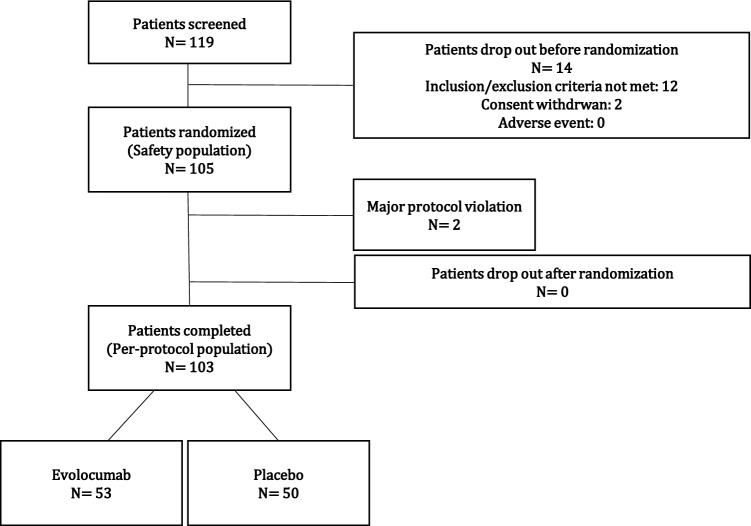
Patient disposition

From April 2019 through July 2021, a total of 119 patients were screened and 103 of them completed the study. Fifty-three patients were randomized to the evolocumab group and fifty to the placebo group (Fig. 2). The average age of the total study population was 66.2 ± 7.7 years and 89.3% of them were male. Office systolic and diastolic BP of the patients were 127 ± 15.8 mmHg and 76 ± 9.1 mmHg, respectively. In the evolocumab group, 84.9% of patients had coronary artery disease, whereas it was 82%% in the placebo group (*p* = 0.695). As defined per study protocol, all patients had statin therapy (72% of the entire population was on at least 20 mg atorvastatin, others on equivalent dose of rosuvastatin, pravastatin or simvastatin) and majority of the patients had either treatment with ACE-inhibitors or AT1-receptor-anatgonists. Clinical characteristics, relevant diagnosis and concomitant medications of the two study groups at baseline are reported in Table 1.

**Table 1:** Clinical characteristics of the patients at baseline

Characteristics	*E* *n* = 53	*P* *n* = 50	*p*-value
Age (years)	66.9 ± 6.9	65.5 ± 8.5	0.371
Gender (% male)	86.8	92.0	0.397
Weight (kg)	87.3 ± 16.3	85.3 ± 12.6	0.480
BMI (kg/m^2^)	28.5 ± 4.6	28.4 ± 4.0	0.945
Office SBP (mmHg)	127 ± 15.7	128 ± 16.2	0.789
Office DBP (mmHg)	75.3 ± 8.5	77.4 ± 9.7	0.239
Office HR (bpm)	62.1 ± 9.8	63.9 ± 9.8	0.358
Fasting Plasma Glucose (mg/dl)	106 ± 21.7	103 ± 12.2	0.393
HbA1c (%)	5.9 ± 0.6	5.8 ± 0.6	0.584
Triglyceride (mg/dl)	113 ± 83.4	112 ± 56.6	0.902
Total cholesterol (mg/dl)	161 ± 25.7	162 ± 29.0	0.857
HDL cholesterol (mg/dl)	51.2 ± 13.0	48 ± 11.8	0.169
LDL cholesterol (mg/dl)	97 ± 17.1	100 ± 21.2	0.428
Kreatinin (mg/dl)	0.95 ± 0.18	0.93 ± 0.17	0.621
eGFR (ml/min)	80 ± 14.0	81 ± 14.3	0.737
Medical history			
Diabetes (n, %)	9, 17	9, 18	0.893
Arterial hypertension (n, %)	43, 81.1	38, 76.0	0.530
Coronary artery disease (n, %)	45, 84.9	41, 82.0	0.695
Non-haemorrhagic stroke or TIA (n, %)	11, 20.8	10, 20.0	0.925
Peripheral artery disease (n,%)	4, 7.5	2, 4.0	0.447
Medications at baseline			
Statin (n, %)	53, 100	50, 100	
Ezetimibe (n, %)	10, 19	11, 22	0.693
AT1 receptor antagonist (n, %)	27, 50.9	26, 52.0	0.916
ACE-inhibitor (n,%)	22, 41.5	17, 34.0	0.437
Mineralocorticoid receptor antagonist (n,%)	4, 7.5	1, 2.0	0.194
Diuretics (n,%)	17, 32.1	16, 32.0	0.994
Betablocker (n,%)	27, 50.9	30, 60.0	0.360
Calcium channel blocker (n,%)	13, 24.5	12, 24.0	0.951

*BMI* body mass index, *SBP* systolic blood pressure, *DBP* diastolic blood pressure, *HR* heart rate, *HbA1c* hemoglobin A1c, *HDL* high density lipoprotein, *LDL* low density lipoprotein, *eGFR* estimated glomerular filtration rate, *AT1* angiotensin 1, *ACE* angiotensin converting enzyme

### Lipid parameters

**Fig. 3:**
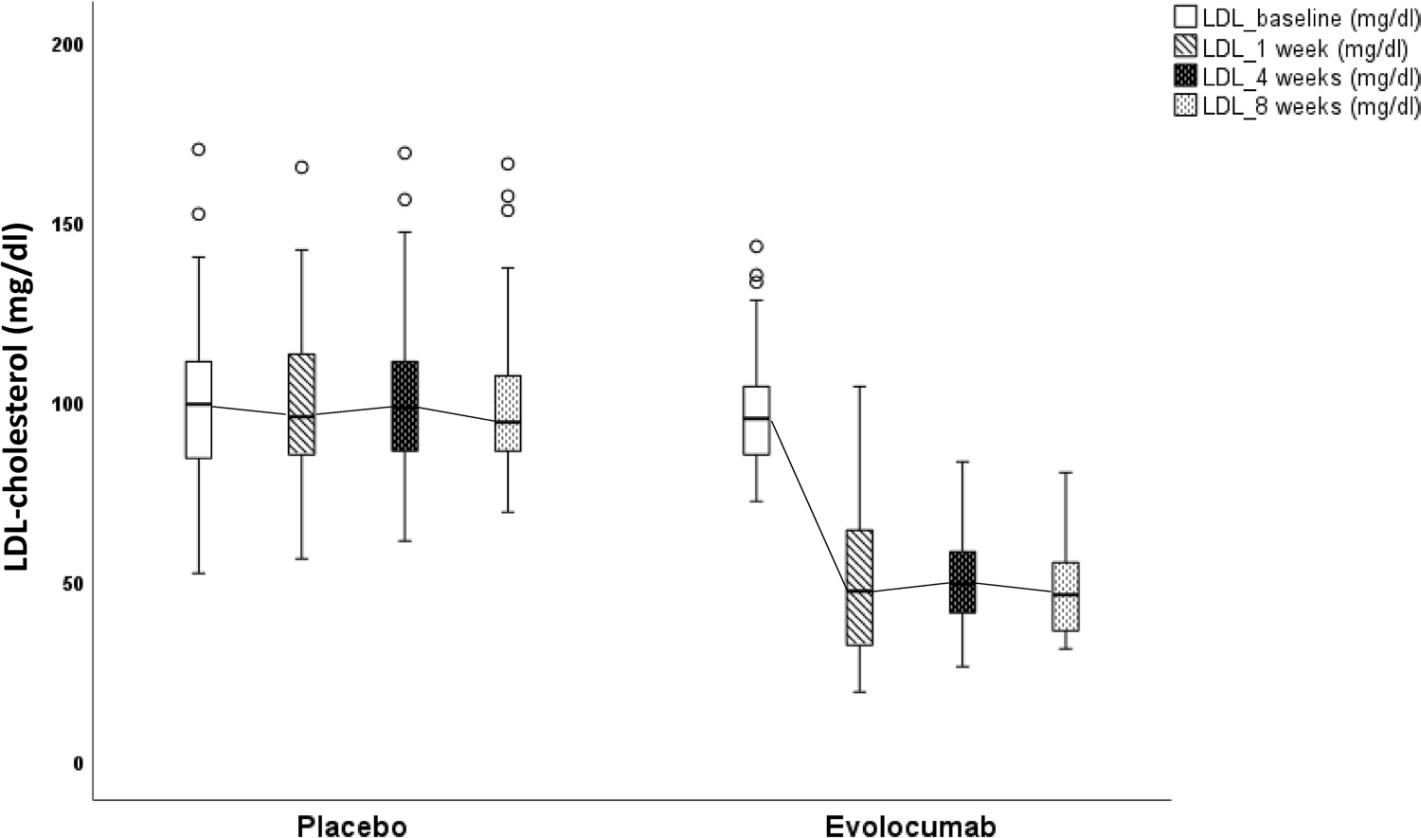
Change in LDL cholesterol level with treatment (placebo/evolocumab). The most reduction in LDL cholesterol levels with evolocumab occurred within one week. The mean reduction in LDL cholesterol in the evolocumab group was maintained over 8 weeks without any further significant reduction after first week

**Fig. 4:**
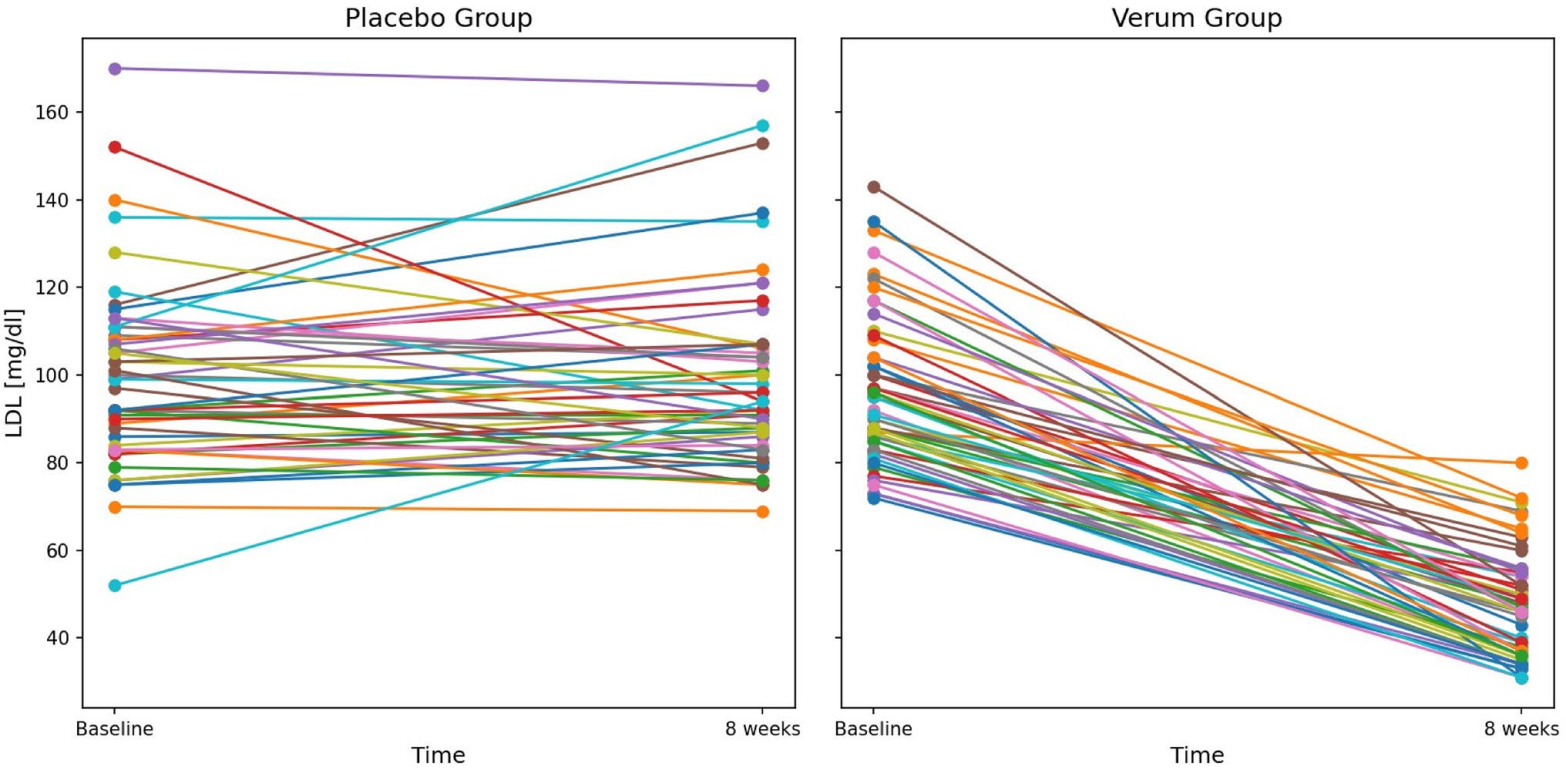
Visualization of the individual variability in LDL-C reduction with treatment (placebo, verum)

Mean LDL-C level prior to randomization was 98 ± 19.1 mg/dl (evolocumab: 97 ± 17.1 mg/dl, placebo: 100 ± 21.2 mg/dl, *p* = 0.428). Reduction in LDL-C levels with evolocumab occurred already within 1 week (Table 2; Fig. 3). Similar reductions could be noticed for total cholesterol and triglyceride levels. The mean reduction in LDL-C, total cholesterol and triglycerides levels in the evolocumab group was maintained over time without any further significant reduction (Fig. 3). The individual variability in LDL-C reduction with treatment (placebo, verum) is shown in Fig. 4. There was a trend of an increase in HDL-C levels in the evolocumab group compared to the placebo group over time within 8 weeks of treatment (*p* = 0.066).

**Table 2:** Effects of Evolocumab (E) vs. Placebo (P) on lipids after 1 and 8 weeks treatment

Lipid parameters	*E* group (Baseline)	*E* group (1 week)	*E* group (8 weeks)	*p*-value E group (Baseline vs 1 week)	*p*-value E group (Baseline vs 8 weeks)	*p* group (Baseline)	*p* group (1 week)	*p* group (8 weeks)	*p*-value P group (Baseline vs 1 week)	*p*-value P group (Baseline vs 8 weeks)	*p*-value ΔE group vs. ΔP group (1 week)	*p*-value ΔE group vs. ΔP group (8 weeks)
Total cholesterol (mg/dl)	161 ± 25.7	106 ± 28.1	105 ± 23.5	** < 0.001**	** < 0.001**	162 ± 29.0	161 ± 27.7	160 ± 30.4	0.539	0.671	** < 0.001**	** < 0.001**
HDL cholesterol (mg/dl)	51 ± 13.0	51 ± 13.2	53 ± 15.2	0.417	0.056	48 ± 11.8	48 ± 12.2	47 ± 11.8	0.817	0.593	0.435	0.066
LDL cholesterol (mg/dl)	97 ± 17.1	49 ± 19.1	47 ± 12.4	** < 0.001**	** < 0.001**	100 ± 21.2	99 ± 20.0	100 ± 21.4	0.364	0.957	** < 0.001**	** < 0.001**
Triglycerides (mg/dl)	113 ± 83.4	99 ± 80.6	87 ± 38.6	**0.012**	**0.004**	112 ± 56.6	114 ± 63.2	120 ± 79.7	0.742	0.289	**0.045**	**0.004**

Bold indicates (*p* < 0.05)

*HDL* high-density lipoprotein, *LDL* low-density lipoprotein

### Endothelial function parameters

**Fig. 5:**
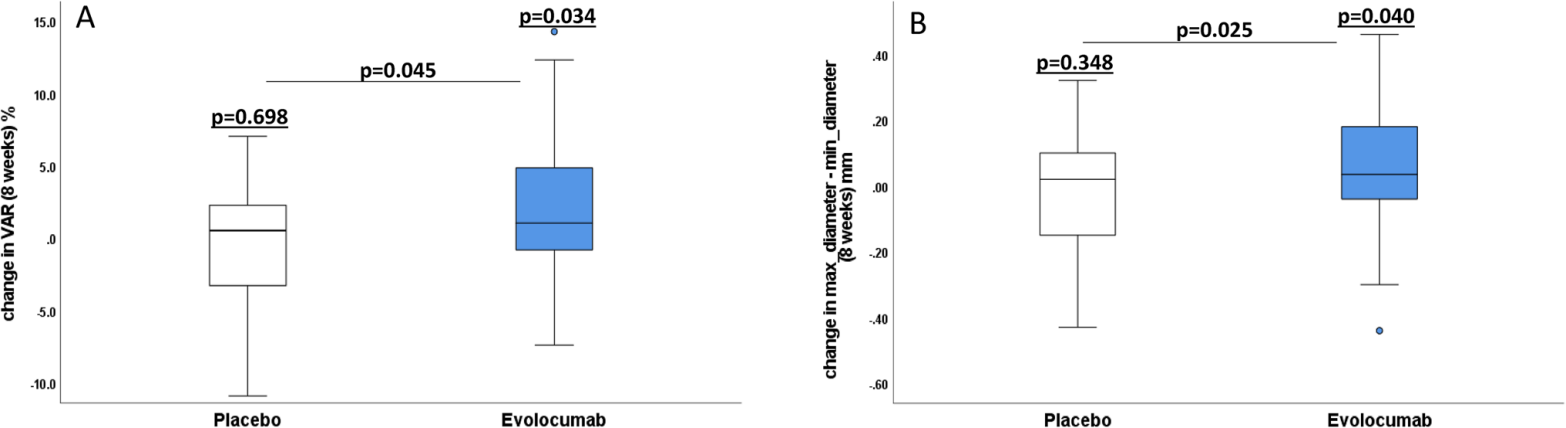
A Change in vasoactive range with treatment (placebo/evolocumab) in 8 weeks. VAR increased after 8 weeks of treatment with evolocumab compared to baseline. The improvement in VAR from baseline at week 8 was significant with evolocumab compared to placebo. B Change in the difference of maximum and minimum diameter of the brachial artery in 8 weeks. The difference of maximum and minimum diameter of the brachial artery increased after 8 weeks of treatment with evolocumab compared to baseline. This improvement from baseline at week 8 was significant with evolocumab compared to placebo

The mean baseline diameter of the brachial artery was similar between the groups (p = 0.618). The change in VAR from baseline to week 8 was significantly different with evolocumab compared to placebo (*p* = 0.045; Fig. 5A; Table 3). Similar results were found for the difference of maximum and minimum diameter of the brachial artery (Fig. 5B; Table 3). However, no change has been noticed in FMD and L-FMC after 8 weeks of treatment with evolocumab. VAR increased after 8 weeks of treatment with evolocumab compared to baseline (*p* = 0.034; Fig. 5A; Table 3). No significant change was observed at 1 and 4 weeks after treatment with evolocumab from baseline versus placebo.

**Table 3:** Effects of Evolocumab (*E*) vs. Placebo (*p*) on vascular function parameters after 8 weeks treatment

Vascular function parameters	E group (Baseline)	E group (8 weeks)	ΔE group (8 weeks)	*p*-value E group (Baseline vs 8 weeks)	*p* group (Baseline)	*p* group (8 weeks)	Δ*p* group (8 weeks)	*p*-value P group (Baseline vs 8 weeks)	Baseline (E group vs P group)	*p*-value ΔE group vs. ΔP group (8 weeks)
Baseline diameter (mm)	4.27 ± 0.5	4.22 ± 0.6	− 0.05 ± 0.4	0.398	4.34 ± 0.5	4.31 ± 0.5	− 0.04 ± 0.3	0.386	0.618	0.849
Minimum diameter (mm)	4.35 ± 0.6	4.26 ± 0.6	− 0.09 ± 0.5	0.230	4.34 ± 0.5	4.32 ± 0.5	− 0.02 ± 0.3	0.659	0.815	0.420
Maximum diameter (mm)	4.44 ± 0.6	4.41 ± 0.6	− 0.04 ± 0.4	0.560	4.50 ± 0.5	4.45 ± 0.5	− 0.04 ± 0.3	0.269	0.709	0.918
Difference of maximum diameter and minimum diameter (mm)	0.09 ± 0.24	0.17 ± 0.17	0.09 ± 0.27	**0.040**	0.16 ± 0.13	0.14 ± 0.16	− 0.02 ± 0.17	0.348	0.093	**0.025**
VAR (mm)	1.98 ± 6.1	4.2 ± 4.3	2.2 ± 7.0	**0.034**	3.8 ± 3.1	3.5 ± 3.9	− 0.24 ± 4.1	0.698	0.096	**0.045**
FMD (%)	4.29 ± 2.8	5.05 ± 3.6	0.76 ± 4.3	0.234	3.92 ± 3.3	4.01 ± 3.1	0.09 ± 3.6	0.865	0.702	0.422
L-FMC (%)	1.61 ± 3.6	0.94 ± 3.7	− 0.67 ± 5.0	0.360	0.10 ± 2.1	0.44 ± 3.1	0.34 ± 3.7	0.549	**0.022**	0.280

Bold indicates (*p* < 0.05)

Baseline diameter (pre-cuff-inflation diameter), minimum diameter (pre-cuff-deflation diameter) and maximum diameter (post-cuff-deflation diameter)

*VAR* vasoactive range, *FMD* flow mediated vasodilation, *L-FMC* low flow-mediated vasoconstriction, bold (p<0.05)

**Fig. 6:**
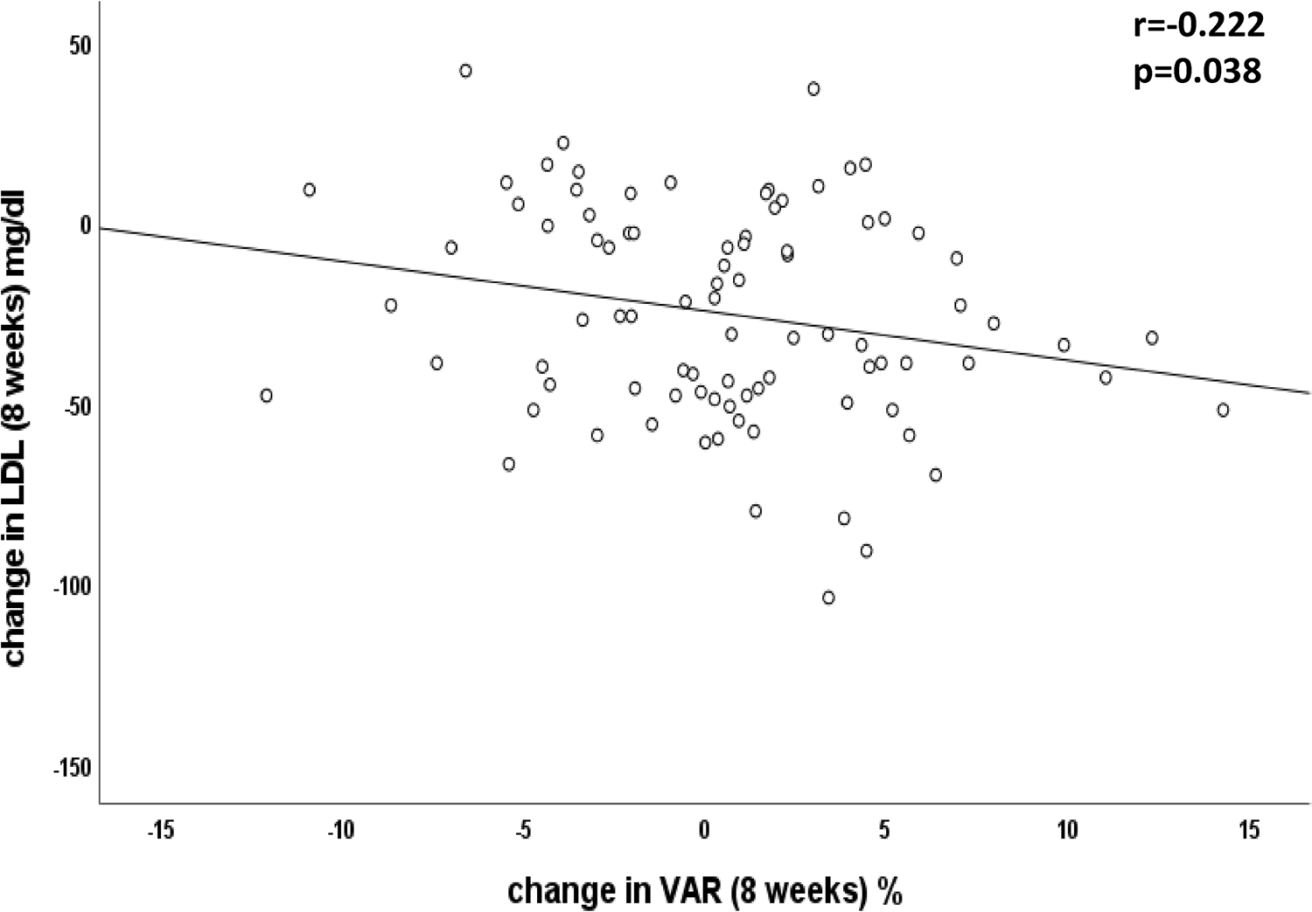
Relationship between change in LDL cholesterol and change in VAR with treatment. In the overall cohort (evolocumab + placebo group), we found a relationship between change in LDL cholesterol and change in VAR with treatment

In the overall cohort, we found a relationship between change in LDL-C and change in VAR with 8 weeks of treatment (*r* = − 0.222, *p* = 0.038, Fig. 6). Similar correlations were found between the change in LDL cholesterol from baseline to 8 weeks of treatment and change in VAR at 1 week (*r* = − 0.212, *p* = 0.047) and 4 weeks of treatment (*r* = − 0.230, *p* = 0.021). No such relationship could be observed between change in LDL-C and other vascular functional parameters.

### Subgroup analyses

In patients with age 67 years or lesser, VAR improved after 8 weeks treatment with evolocumab compared to baseline (*p* = 0.006). This improvement was different compared to patients with age greater than 67 years (*p* = 0.013). Similar changes was found in the difference of maximum and minimum diameter of the brachial artery (*p* = 0.006) with evolocumab treatment in the younger age group, which was also different between both age groups (*p* = 0.014). In patients with higher LDL-C level at baseline (> 95 mg/dl), changes in VAR and the difference of the maximum and minimum diameter was observed (*p* = 0.042 and *p* = 0.034, respectively) with evolocumab treatment. In patients with lower systolic BP (≤ 125 mmHg), only changes in VAR was observed with evolocumab treatment (*p* = 0.049). We found no change in endothelial function parameters with treatment based on HbA1c or achieved LDL-C levels.

### Safety

During 12 weeks of study period no significant group differences were noticed in the total rates of adverse events, serious adverse events, or adverse events thought to be related to the study agent. Twelve patients dropped out of the study before randomization due to the inclusion or exclusion criteria of the study and two have withdrawn their consent. In total, four serious adverse events occurred during the study. None of these serious adverse events were related to study medications and all patients recovered.

## Discussion

Our trial attempted to answer the question whether the PCSK-9 inhibitor evolocumab is able to improve vascular function in patients with CV disease. The main result of our study is that endothelial function as assessed by the total vasomotor responsiveness (VAR) and the difference of maximum diameter and the minimum diameter, improved in the evolocumab treated group after 8 weeks of treatment compared to the placebo group and to baseline. In the overall cohort, we found a relationship between change in LDL-C and change in VAR with treatment. Recently, Königstein et al. demonstrated that the parameter VAR has the highest predictive power of CV risk compared with FMD and L-FMC [12]. The UNEX system we used in our study overcomes major limitations of previous hand held ultrasound systems [16]. This semiautomatic system tracks the brachial artery diameter automatically and in real time, adhering to expert recommendations for the assessment of FMD to reduce inter-observer variability and improve data comparability between studies [14]. Of note, we observed an improvement in endothelial function with evolocumab treatment in patients with established cardiovascular disease. In addition, this improvement of endothelial function even occurred in this specific study population on obligatory statin therapy and majority of the patients were under treatment with either ACE-inhibitors or AT-1-receptor-blockers, both known to improve endothelial function [18, 19]. Previously, our group has shown that statin treatment improves vascular function in patients with elevated LDL-C, which at least in part serves to explain the beneficial effects of statins on CV outcomes [20, 21]. Moreover, we were able to show that an improvement in endothelial function during statin therapy is linked with an improvement of pulse wave reflection [22]. With the present randomized placebo-controlled study, we are able to document an improvement of endothelial function with evolocumab. Our results contribute to the mechanistic explanations why lower incidence of the CV composite endpoint (CV death, myocardial infarction, stroke, hospitalization for unstable angina, or coronary revascularization) has been demonstrated in the FOURIER study. In support to our results, a non-randomized pilot study has demonstrated a significant improvement in coronary endothelial function with evolocumab treatment in patients with HIV and dyslipidemia [23].

Changes in FMD and L-FMC after 8 weeks treatment with evolocumab compared to placebo, were not significantly different, but parameters most recently introduced in vascular medicine, such as VAR, which is more sensitive and also predictive for CV events was found to be different between evolocumab and placebo treatment groups [12]. We believe that FMD and L-FMC are less sensitive parameters, in particular in patients with established cardiovascular disease. The measurement of FMD or L-FMC alone may not adequately reflect endothelial responsiveness towards altered hemodynamic stimuli [24, 25]. FMD measures the nitric oxide-dependent part of endothelial function and L-FMC appears to be nitric oxide-independent [26, 27]. VAR, a composite of vascular dilation and vascular constriction reflects the maximum possible changes of a vessel and might be the most sensitive parameter of the vascular ability to change the diameter via changes in vascular tone [12]. The vasoconstrictive response alone as well as the vasodilatory response alone reflects just part of the ability of a vessel to change the vascular tone and thereby of the vascular functional capacity. We previously demonstrated that the contribution of nitric oxide to FMD in healthy men is only 40%, assessed with the same reliable semiautomatic ultrasound device used in this study [15]. The contribution of the nitric oxide to FMD in patients with established cardiovascular disease is unknown. In earlier stages of cardiovascular disease, an improvement in FMD has been shown in patients with increased CV risk with evolocumab treatment. However, this was a non-randomized non-controlled pilot study with a small sample size of 14 patients only [28]. In another study, the addition of evolocumab along with empagliflozin showed progressively improved FMD, increased bioavailability of nitric oxide metabolites and reduced oxidative stress estimated by blood isoprostane levels in patients with type 2 diabetes. However, the majority of patients in this study were without CV disease [29].

Several studies have provided strong support for the concept that lower LDL-C levels are the key to achieve better outcomes[30, 31], and that it is possible to achieve these on top of statin therapy (despite of the much debated potential “pleiotropic” effects of statins) [3, 32]. In our study, we found a relationship between change in LDL-C and change in VAR with treatment, but no such relationship was found with achieved LDL-C. However, this relationship could be found only in the overall cohort considering evolocumab and placebo treatment groups together. This suggests that the reduction of LDL-C might not be the only mechanism leading to the improvement in endothelial function [33]. In the FOURIER-OLE, there was a monotonic relationship between lower achieved LDL-C levels and a lower risk of the CV composite endpoints [34].

Rapid improvement of endothelial and large artery function is considered to be particularly important in patients at high CV risk, such as those that have suffered a recent CV event. Improvement of basal NO activity in the renal circulation and NO-dependent vasodilation of the peripheral vasculature occurs already 3 days after initiating treatment of statins [20–22]. In our study, 8 weeks of treatment was needed to improve the endothelial functional parameters, in particular VAR. A relationship between the change in LDL-C from baseline to 8 weeks of treatment and change in VAR at all time points has been found. In our exploratory analysis, no change in these endothelial function parameters was noticed 1 week and 4 weeks after treatment. In contrast, a mean reduction of LDL-C of 47 ± 13.1 mg/dl was noticed already after 1 week of treatment with evolocumab. Further reduction of LDL-C during the treatment period was not observed. After 8 weeks of treatment with evolocumab, the mean LDL-C reduction was 49 ± 17.3 mg/dl. In addition, similar results have been noticed with respect to total cholesterol and triglycerides. This distinction between rapidness of improvement of endothelial function and LDL decrease may be related to the effect that improvement of endothelial function in patients with established cardiovascular disease take longer period than one week of treatment duration. If other less sensitive endothelial functional parameters would have improved with longer period of treatment is unknown. A delay between the onset of LDL-C lowering and the emergence of clinical risk reduction has been well described in studies of statins and ezetimibe [35, 36]. This delay may be due to the time needed for changes in the anatomical structure of the vessel wall, such as alterations in elastin, collagen content and fibrotic tissue. Pulse wave velocity could be a suitable parameter to assess such long-term effects.

In the subgroup analysis, in patients with age less than 67 years, we found clear improvement in the parameters of endothelial function. Similar results have been demonstrated in patients with lower systolic BP and patients with higher baseline LDL-C. These subgroup analysis demonstrated that improvement of vascular function can be achieved by initiating evolocumab treatment particularly in young patients with lesser vascular target organ damage.

No safety signal was noted in our study. Four patients were admitted to hospital during the study due to congestive heart failure with dyspnea, hypertensive crisis, NSTEMI and surgical removal of hematoma from the pretibial region left caused by fall from bicycle. None of these serious adverse events (SAE) were related to study medication and all patients recovered. There was no drop-out due to SAE over the course of the study period. No adverse events raised concerns about the safety of evolocumab.

Our study has strengths and limitations. We are the first, demonstrating in a randomized controlled study an improvement in endothelial functional parameters with evolocumab treatment in a cohort similar to the FOURIER cohort. However, this was a single-center study with an overall small sample size, anyway designed to be a mechanistic study. The major limitation was a relatively short duration of the study with last assessment of the vascular function 8 weeks after randomization. Thereby, we are not able to make any statements regarding how long the improvement in vascular functional parameters would sustain.

## Conclusion

With this clinical trial, we observed that endothelial function could be improved with evolocumab treatment in high-risk patients with preexisting CV disease and on statin therapy. Our results contribute to the mechanistic explanation why lower incidence of the CV composite endpoint has been demonstrated in the FOURIER study.

## Data Availability

The datasets used and/or analyzed during the current study are available from the corresponding author on reasonable request.

## Abbreviations

ACE: Angiotensin converting enzyme
AT1: Angiotensin 1
BMI: Body mass index
BP: Blood pressure
CV: Cardiovascular
eGFR: Estimated glomerular filtration rate
FMD: Flow-mediated vasodilatation
HDL-C: High-density lipoprotein cholesterol
HR: Heart rate
LDL-C: Low-density lipoprotein cholesterol
L-FMC: Low flow mediated vasoconstriction
NO: Nitric oxide
NSTEMI: Non-ST-elevation myocardial infarction
OLE: Open label extension
PCSK9: Protein convertase subtilisin/kexin type 9
SAE: Severe adverse event
SC: Subcutaneous
SD: Standard deviation
TIA: Transient ischemic attack
VAR: Vasoactive range
